# Evaluation of *Bifidobacteria* and *Lactobacillus* Probiotics as Alternative Therapy for *Salmonella typhimurium* Infection in Broiler Chickens

**DOI:** 10.3390/ani10061023

**Published:** 2020-06-12

**Authors:** Hanem El-Sharkawy, Amin Tahoun, Amira M. Rizk, Tohru Suzuki, Walid Elmonir, Eldsokey Nassef, Mustafa Shukry, Mousa O. Germoush, Foad Farrag, May Bin-Jumah, Ayman M. Mahmoud

**Affiliations:** 1 Department of Poultry and Rabbit Diseases, Faculty of Veterinary Medicine, Kafrelsheikh University, Kafrelsheikh 33511, Egypt; hanem_amin@yahoo.com; 2 Department of Animal Medicine, Faculty of Veterinary Medicine, Kafrelsheikh University, Kafrelsheikh 33511, Egypt; 3 Faculty of Applied Biological Sciences, Gifu University, 1-1 Yanagido, Gifu 501-1193, Japan; suzuki@cc.gifu-u.ac.jp; 4 Department of Bacteriology, Mycology and Immunology, Faculty of Veterinary Medicine, Benha University, Benha 13511, Egypt; dr_az80@yahoo.com; 5 Department of Hygiene and Preventive Medicine, Faculty of Veterinary Medicine, Kafrelsheikh University, Kafrelsheikh 33511, Egypt; walid.elmonir@gmail.com; 6 Department of Nutrition and Clinical Nutrition, Faculty of Veterinary Medicine, Kafrelsheikh University, Kafrelsheikh 33511, Egypt; dsokeynassef@yahoo.com; 7 Department of Physiology, Faculty of Veterinary Medicine, Kafrelsheikh University, Kafrelsheikh 33511, Egypt; mostafa.ataa@vet.kfs.edu.eg; 8 Biology Department, College of Science, Jouf University, Sakaka 2014, Saudi Arabia; mogermoush@ju.edu.sa; 9 Department of Anatomy and Embryology, Faculty of Veterinary Medicine, Kafrelsheikh University, Kafrelsheikh 33511, Egypt; foad.farrag@yahoo.com; 10 Department of Biology, College of Science, Princess Nourah bint Abdulrahman University, Riyadh 84428, Saudi Arabia; mnbinjumah@pnu.edu.sa; 11 Physiology Division, Department of Zoology, Faculty of Science, Beni-Suef University, Beni-Suef 62514, Egypt

**Keywords:** *Salmonella typhimurium*, *Bifidobacteria*, *Lactobacilli*, probiotics, antibiotic alternatives, broiler chickens

## Abstract

**Simple Summary:**

*Salmonella* is an important foodborne pathogen that represents a very critical threat to poultry industry worldwide. This study concerns an important aspect of human food and health problem by treating a common zoonotic bacterial disease in poultry industry. Owing to the increased resistance to antibiotics among *Salmonella* *enterica* serotypes, we aimed to explore the beneficial effects of different probiotics strains as alternative sources of protection against infection in broiler chickens. Three probiotic strains *Lactobacillus* (*Lacticaseibacillus*) *casei* ATTC334, *Bifidobacterium breve* JCM1192 and *Bifidobacterium infantis* BL2416) improved body weight gain and prevented the deleterious effects and mortality induced by *Salmonella* infection in chicks through different mechanisms, including competitive exclusion and the promotion of cytokines’ release.

**Abstract:**

Chicken *Salmonella enterica* serovars are enteric bacteria associated with massive public health risks and economic losses. There is a widespread antimicrobial resistance among *S.* *enterica* serotypes, and innovative solutions to antibiotic resistance are needed. We aimed to use probiotics to reduce antibiotic resistance and identify the major probiotic players that modify the early interactions between *S.* *enterica* and host cells. One-day-old cobb broiler chicks were challenged with *S. typhimurium* after oral inoculation with different probiotic strains for 3 days. The adherence of different probiotic strains to Caco-2 intestinal epithelial cells was studied in vitro. *Lactobacillus* (*Lacticaseibacillus*) *casei* ATTC334 and *Bifidobacterium breve* JCM1192 strains attached to Caco-2 cells stronger than *B. infantis* BL2416. *L. casei* ATTC334 and *B. breve* JCM1192 reduced *S. typhimurium* recovery from the cecal tonsils by competitive exclusion mechanism. Although *B. infantis* BL2416 bound poorly to Caco-2 epithelial cells, it reduced *S. typhimurium* recovery and increased IFN-γ and TNF-α production. *L. casei* ATTC334, *B. breve* JCM1192 and *B. infantis* BL2416 improved body weight gain and the food conversion rate in *S. typhimurium*-infected broilers. *B. longum* Ncc2785 neither attached to epithelial cells nor induced IFN-γ and TNF-α release and consequently did not prevent *S. typhimurium* colonization in broiler chickens. In conclusion, probiotics prevented the intestinal colonization of *S. typhimurium* in infected chickens by competitive exclusion or cytokine production mechanisms.

## 1. Introduction

*Salmonella* is one of most important pathogenic bacteria implicated in foodborne bacterial outbreak throughout the world. In the last two decades, *Salmonella* represented the major causative agent of foodborne gastroenteritis [1,2]. *Salmonella* threaten poultry welfare and exert a substantial economic burden owing to their ability to migrate to systemic sites, causing fowl typhoid and paratyphoid. *S. typhimurium* has been documented to be the major cause of avian salmonellosis in broiler chickens [3]. The levels of *Salmonella* infection in poultry in Egypt are amongst the highest in the world [4]. The outbreaks of human salmonellosis are originating from key food production animals. This food associated zoonosis is linked with a wide variety of foods of animal origin; however, the main source of human infection is poultry meat and eggs [5,6].

Host-bacterial interactions have a profound influence on health and disease. The chicken gastrointestinal tract (GIT) is inhabited by multiple commensal bacteria that aid in the digestion of food and production of vitamins and short-chain fatty acids that play vital roles in poultry physiology and disease [7,8,9]. The GIT pathogens such as *S. enterica* measure carbon sources as an important signal to regulate the expression of its virulence genes. *Salmonella* senses whether it is in a gluconeogenic versus a glycolytic environment, as well as fluctuations in sugar levels to fine tuned regulation of its virulence repertoire [10]. A number of studies have defined that the use of probiotics was associated with resistance to a very wide range of infections [11,12], in particular, systemic salmonellosis in poultry [13,14]. These probiotics oppose *Salmonella* colonization by reducing the pH by production of short-chain fatty acids to a level that is not suitable for *Salmonella* survival [7]. Furthermore, probiotic bacteria can interfere with the gene expression pathways of pathogenic bacteria, rendering the pathogen unable to colonize and cause disease [15]. These probiotics also manipulate rapid immune mechanisms by intestinal cells to produce antimicrobial peptides that lead to more effective control strategies to reduce the impact of *Salmonella* infection [16]. The antimicrobial peptides kill bacteria by forming pores in the membrane; however, these peptides play a key role in immune response through the recruitment of neutrophils to sites of infection [17]. In addition, the presence of these probiotics as feed supplements for poultry can promote immunomodulatory activities, mitigate lipopolysaccharide (LPS)-induced tumor necrosis factor (TNF)-α factor (LITAF), interleukin (IL)-1β, IL-6 and IL-12, and increase the IL-10 during a challenge with *Salmonella*. This innate immune response links to and directs the adaptive immune system, and thus translates into more efficacious immunomodulatory mechanisms that reduce the colonization of *Salmonella* [13,18].

Antibiotic resistance is a major problem in the treatment of bacterial infections. *Salmonella* is becoming resistant to several antibiotics that lead to treatment failure [19]. In addition to improvements in biosecurity and husbandry practices, using probiotic bacteria as an immune-modulator and competitor with pathogenic bacteria for colonization sites is another important procedure to reduce the prevalence of foodborne pathogens, like *Salmonella*, in the reservoir species especially broiler chicken [20], thereby limiting their entry to the human food chain. In this regard, we aimed to develop innovative interventions that could serve as effective antibiotic alternatives, especially for treatment of salmonellosis in chicken farms. We studied the adherence of different *Lacticaseibacillus* (*Lactobacillus*) and *Bifidobacterium* strains to Caco-2 intestinal epithelial cells and evaluated their beneficial effects in *S. typhimurium*-infected broiler chickens.

## 2. Materials and Methods

### 2.1. S. typhimurium Culture and Determination of Colony Forming Unit (CFU)

A primary poultry isolate of *S. typhimurium* was used for these experiments as described previously [4]. *S. typhimurium* was incubated at 37 °C for 24 h and passed every 12 h. The bacterial cells were washed three times in sterile saline by centrifugation at 4000 rpm. The culture was subjected to 10-fold serial dilution in phosphate-buffered saline (PBS) and concentrations of *S. typhimurium* were determined by spread-plating each dilution on XLD agar (Oxoid, Basingstoke, UK).

### 2.2. Probiotics Culture

*Lacticaseibacillus casei* ATTC334, *Bifidobacterium breve* JCM1192, *B. longum* Ncc2785 and *B. infantis* BL2416 were provided by Professor Tohru Suzuki (Faculty of Applied Biological Sciences, Gifu University, Gifu, Japan). The probiotic isolates were cultured in MRS broth medium and then diluted in reconstituted powdered skimmed milk to an expected concentration of 10^8^ CFU/mL. Actual CFUs administered per chick from each experiment were determined retrospectively by serially diluting the overnight culture of the probiotic strains, and plating onto MRS agar (BD Diagnostics, Franklin Lakes, NJ, USA) to determine the number of bacteria in culture as CFU.

### 2.3. Adhesion of Probiotic Strains to Caco-2 Cells

Caco-2 intestinal epithelial cells (American Type Culture Collection) were seeded for 2 weeks on minimum essential medium (MEM; Sigma, St. Louis, MO, USA) supplemented with 10% fetal bovine serum (FBS), 1% glutamine and 1% penicillin/streptomycin (100 U/mL) at 37 °C in 5% CO_2_ and moisture to allow cellular differentiation. Caco-2 cells were then seeded onto 20 mm coverslip coated with mouse collagen IV (Trevigen, Gaithersburg, MA, USA) in 12-well plate at 10^5^ cells per well 24 h before being challenged with the bacteria. The adhesion of probiotics strains to Caco-2 cells was evaluated as previously described [21]. Briefly, the stationary phase lactic acid bacteria (LAB) strains grown in MRS were diluted in MEM to an optical density of 0.9 at 600 nm. An aliquot of this culture was used to challenge a confluent Caco-2 intestinal epithelial cells at a multiplicity of infection of ~100 bacteria to epithelial cell in triplicate. The binding studies were performed at 37 °C, 5% CO_2_ and 80% humidity for 1 h. For direct visualization of adherent bacteria, the cells were fixed in 99.8% methanol for 10 min at room temperature then stained with Giemsa stain (Muto Pure Chemicals, Tokyo, Japan) at 1:10 dilution for 20 min. A total of 100 cells were examined under the light microscope and the number of adhered bacteria to each cell was counted in 20 randomly selected fields.

### 2.4. Birds and Housing

One hundred and twenty apparently healthy, one-day-old Cobb broiler chicks were enrolled in the experiment. The chicks were housed in six separate pens with sawdust bedded floors. Each pen was equipped with a feeder, drinker and brooder. The house was equipped with fans, air suction and a thermometer to control temperature and humidity. The experiment was conducted at the Poultry Research Unit and approved by the Animal Care and Use Committee of Faculty of Veterinary Medicine, Kafrelsheikh University (Egypt).

The birds were fed ad libitum and feed intake was measured daily at 08:00 h in triplicates as feed offered refusals. The birds were blocked by body weight, with each block assigned randomly to one of the six treatments. Starter, grower and finisher diets were formulated according to nutrients specification of Cobb broiler requirements (Table 1). Birds were fed starter diet from 1–21 days, grower diet from 22–28 days, and finisher diet from 29–35 days.

Premix supplies vitamin A 12,000 Iu, vitamin D 5000 Iu, vitamin E 50 mg, vitamin K3 3 mg, vitamin B1 3 mg, vitamin B2 8 mg, nicotinic acid 30 mg, pantothenic acid 15 mg, vitamin B6 4 mg, vitamin B12 0.016 mg, folic acid 2 mg, biotin 0.2 mg, manganese 120 mg, zinc 100 mg, iron 40 mg, copper 16 mg, iodine 1.25 mg, selenium 0.3 mg) per 1 kg diet. The diet composition was calculated according to the National Research Council (NRC) 1994 feed composition table [22].

### 2.5. Experimental Design

The experiment was designed according to the method described by Akbari et al. [18], with some modifications. One-day-old broiler chicks were obtained from a local *Salmonella*-free hatchery. Immediately after arrival, the chicks and their boxes were sampled for the presence of *Salmonella*. The birds were divided randomly into six groups (*n* = 20) as described in Table 2. Half of the birds of each group were scarified the next day of *S. typhimurium* challenge and the others continued to be treated with probiotics every day until the end of the experiment to check the effect of probiotics on body weight gain and food conversion ratio (FCR). The birds were inoculated orally with 1 mL containing 2 × 10^9^ CFU from each candidate of probiotic bacteria via oral gavage for 3 days and then challenged with 1 mL containing 10^8^ CFU wild-type strain of *S. typhimurium* via oral gavage. Chicks were scarified 24 h after challenge with *S. typhimurium* and cecal tonsils were aseptically removed and placed into sterile tubes containing selenite-F broth. The samples were incubated overnight at 37 °C and streaked on XLD agar, incubated for 16 h at 37 °C and the presence or absence of *S. typhimurium* colonies was documented. The cecum from each chicken was removed aseptically and the contents were homogenized and diluted with sterile PBS (10% *w*/*v*) and the spread plated onto XLD plates to enumerate *S. typhimurium*. The plates were incubated at 37 °C for 16 h, and the CFU of *S. typhimurium* per cecal pair were determined.

To investigate the clinical signs and lesions, the birds were observed daily from the start of challenge to the end of the experiment.

### 2.6. Determination of Serum IFN-γ and TNF-α

Blood samples were collected, and serum was separated for the assay of IFN-γ and TNF-α levels using specific ELISA kits supplied by MyBioSource (San Diego, CA, USA).

### 2.7. Statistical Analysis

The results were presented as mean ± standard deviation (SD). Statistical analysis was performed using one-way ANOVA followed by Tukey’s test on GraphPad Prism 7 and differences were indicated to be statistically significant at *p* < 0.05.

## 3. Results

### 3.1. LAB-Epithelial Adherence

**Figure 1:**
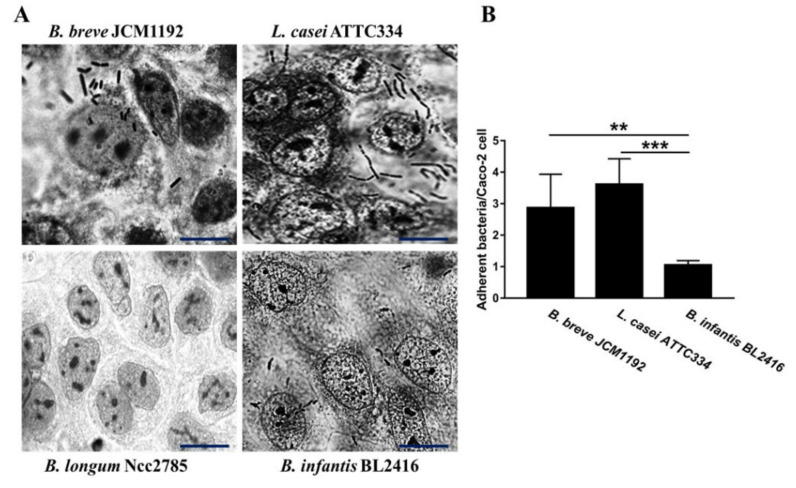
Comparison of different probiotics interactions with Caco-2. (**A**) Images acquired using a phase contrast microscopy showing the adherence of bacterial strains to Caco-2 cells. Confluent monolayers of the Caco-2 cells were challenged with *B. breve* JCM1192, *L. casei* ATTC334, *B. longum* Ncc2785 or *B. infantis* BL2416 strains at a multiplicity of infection of ~100 (bacteria to epithelial cell) for 60 min. The cells were washed 3 times with PBS, fixed with methanol and stained with Giemsa stain. [Scale bar = 20 µm] (**B**) A total of 100 cells were examined under the light microscope and the number of bacteria adhered to each cell were counted in 20 randomly selected fields. The number of attached *B. breve* JCM1192 *B. L. casei* ATTC334, *infantis* BL2416 cells per Caco-2 cell were 2.9 ± 1.1, 3.6 ± 0.8 and 1.05 ± 0.1 (nucleus ± SD) respectively; but no adherent bacteria were observed in *B. longum* Ncc2785. Data are represented as mean ± SD. ** *p* < 0.01 and *** *p* < 0.001. The experiment was repeated three times (*n* = 3).

To examine whether there are differences in the interaction of LAB with Caco-2 epithelial cells, a panel of *Bifidobacteria* spp. and *L. casei* ATTC334 strains were used in the adherence assays. Using previously established criteria to define adherence phenotypes [21], *L. casei* ATTC334, *B. breve* JCM1192 and *B. infantis* BL2416 exhibited significant adherence, whereas *B. longum* Ncc2785 did not attach to Caco-2 cells (Figure 1). Quantitative analysis of the bacteria adherent demonstrated that *L. casei* ATTC334 and *B. breve* JCM1192 strains adheres significantly to Caco-2 cells relative to *B. infantis* BL2416 (Figure 1).

To investigate whether the binding of the probiotic strains to epithelial cells in vitro could affect *Salmonella* infection in vivo through their competition for binding sites and interaction with host immune cells, chicks were inoculated orally with the four different strains of probiotic bacteria for 3 days before challenge with wild type *S. typhimurium*. The results indicated that each probiotic strain which has the ability to bind to Caco-2 epithelial cells in vitro was sufficient to cause a significant reduction in *S. typhimurium* colonization to cecal tonsils with *L. casei ATTC334* and *B. breve* JCM1192 strains being the most effective (Table 3). Treatment of the broiler chicks with *B. breve* JCM1192, *L. casei* ATTC334 or *B. infantis* BL2416 before *S. typhimurium* challenge significantly reduced cecal tonsils colonization by 20%, 10% and 30%, respectively. In addition, *B. breve* JCM1192, *L. casei* ATTC334 or *B. infantis* BL2416 resulted in a significant reduction in *S. typhimurium*-cecal recovery. The results showed 26.4 ± 8.067 (×10^4^), 17.18 ± 3.45 (×10^4^) and 22.61 ± 6.65 (×10^4^) *S. typhimurium*-cecal recovery in chicks treated with *B. breve* JCM1192, *L. casei* ATTC334 or *B. infantis* BL2416, respectively, when compared with the control (230.0 ± 4.14 (×10^4^)) and *B. longum* Ncc2785-treated (179.03 ± 7.81 (×10^4^)) groups as outlined in Table 3.

### 3.2. Clinical Signs and Postmortem (PM) Lesions

Birds infected with *S. typhimurium* alone or with *B. longum* Ncc2785 showed variable clinical signs including inappetence, depression, ruffled feathers and pasty diarrhea started at the 3rd day post-challenge. The mortality rate was 30% in the control positive group and 20% in the group treated with *B. longum* Ncc2785. In the PM lesions in the birds in the above two groups, the liver was enlarged and friable with observed necrotic foci and the gall bladder was distended. There were also lesion of pericarditis and splenomegaly. All the deaths occur from day 3 to 7 after challenge with *S. typhimurium*. Birds treated with *B. breve* JCM1192, *L. casei* ATTC334 or *B. infantis* BL2416 then infected with *S. typhimurium* did nt exhibit clinical signs or mortality.

### 3.3. Probiotics Improve Growth Performance in S. typhimurium-Infected Broiler Chickens

**Figure 2:**
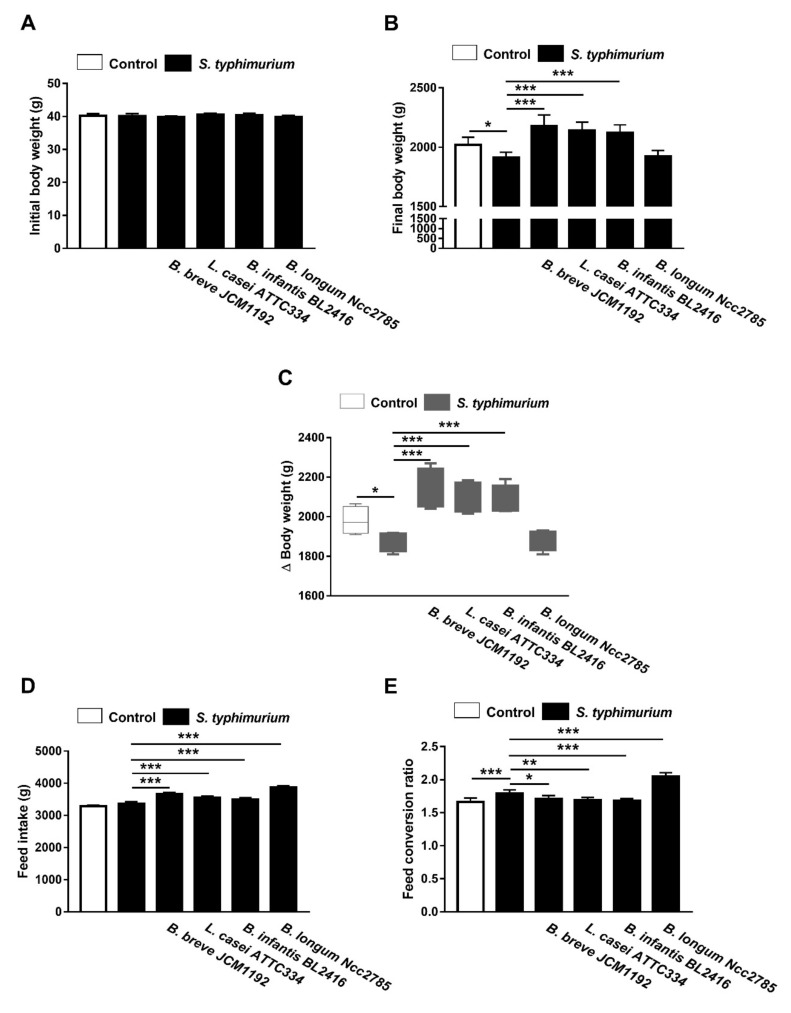
Effect of *Bifidobacteria* and *Lacticaseibacillus* probiotics on growth performance of broiler chickens challenged with *S. typhimurium.* Probiotics improved body weight gain (**A**–**C**), feed intake (**D**) and feed conservation ratio (**E**). Data are mean ± SD, (*n* = 10). * *p* < 0.05, ** *p* < 0.01 and *** *p* < 0.001.

The results of growth performance are shown in Figure 2. Chicks infected with *S. typhimurium* showed a decrease in final body weight at day 35 (5.2%) and body weight gain (BWG; 5.3%) as compared to the control. Oral supplementation of *B. longum* Ncc2785 failed to improve the growth rate of the control chicks and was nearly similar to the infected non-treated chicks. Chicks treated with *L. casei* ATTC334, *B. breve* JCM1192 or *B. infantis* BL2416 had a significantly higher growth rate than the control chicks (Figure 2).

*S. typhimurium* showed that infected chicks had poor FCR (7.83%) compared to the control chicks, although there was non-significant difference in feed intake. Treatment with *B. longum* Ncc2785 offended FCR (23.49%) compared to the control chicks. On the other hand, treatment with *L. casei* ATTC334, *B. breve* JCM1192 or *B. infantis* BL2416 achieved similar FCR when compared to the control chicks (Figure 2).

### 3.4. Effect of Probiotics on Serum TNF-α and IFN-γ Levels in and S. typhimurium-Infected Broiler Chickens

**Figure 3:**
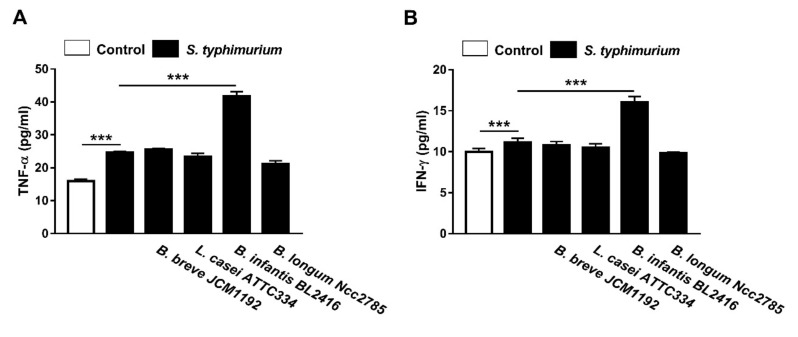
Effect of oral administration of different probiotics on (**A**) TNF-α and (**B**) IFN-γ levels in the peripheral blood of broiler chickens. Data are mean ± SD, (*n* = 10). *** *p* < 0.001.

We investigated whether the exposure to probiotics and *S. typhimurium* triggers cytokines secretions in the peripheral blood of challenge broiler chickens. Serum TNF-α (Figure 3A) and IFN-γ (Figure 3B) levels were investigated by ELISA. TNF-α was increased significantly in the serum of *S. typhimurium*-infected chicks when compared with the control group. Although *B. breve* JCM1192, *L. casei* ATTC334 and *B. longum* Ncc2785 strains produced non-significant changes, *B. infantis* BL2416 significantly increased serum TNF-α in *S. typhimurium*-infected chicks (Figure 3A).

Similarly, serum IFN-γ was significantly increased in *S. typhimurium*-infected chicks received *B. infantis* BL2416 (Figure 3B), whereas other strains did not exert significant effects.

## 4. Discussion

The ability of bacteria to bind to intestinal epithelial cells has been considered as one of the most important selection properties for probiotic strains [23,24]. It is apparent that probiotics bacterial adhesions to the intestinal epithelial cells facilitates their attachments during intestinal colonization by avoiding bacterial removal by peristalsis and providing a competitive exclusion advantage, supporting its role in the ecosystem as an anti-infection strategy [25,26]. Our results indicated that *L. casei ATTC334* and *B. breve* JCM1192 strains exhibited significant adherence when compared with *B. infantis* BL2416 and *B. longum* Ncc2785. Therefore, these strains may be associated with a greater capacity to be used as a probiotic. The attachment of probiotic to epithelial cells is a process that involves manipulation of the pathogenic bacterial infection [27]. Our results suggest that a reduction in *S. typhimurium* colonization to cecal tonsils with *L. casei ATTC334* and *B. breve* JCM1192 strains were due to their ability to attach to epithelial cells, as shown by the in vitro study. *B. longum* Ncc2785 strain was not able to attach to Caco-2 cells in vitro, which could be explained in terms of the composition of surface polysaccharide that can prevent adhesion by inhibiting the expression of some bacterial factors, such as fimbriae that aid bacterial adherence [21].

Previous studies have shown that cytokines are implicated in the clearance of *Salmonella* from the gut [28,29]. IFN-γ is a major factor that has been implicated in many of the intestinal and systemic immunological consequences [30,31]. Its induced increase in gut motility during an infection is likely to enhance the clearing and fecal shedding of pathogens [32]. Given that enhanced phagocytosis could arise through increased production of IFN-γ [33], we examined the effect of all probiotic strains used in this study on serum IFN-γ. The results indicated that only *B. infantis* BL2416 was able to increase IFN-γ secretion in chicks. This probiotic strain reduced the recovery rate of *S. typhimurium* from cecal tonsils that may occur as a result of synergy between the probiotics and host macrophages. These interactions and the colonization of probiotics to the host epithelial cells caused a trend towards further reduction rates of *Salmonella* due to competition for the colonization sites. Similar results have been reported in previous studies [34,35,36,37].

Moreover, *B. infantis* BL2416 was the only probiotic strain that increased serum TNF-α in *S. typhimurium*-induced chicks. TNF-α is a major factor of inflammation in birds and is produced by monocytes, tissue macrophages, enterocytes, and other cells [32]. Owing to its ability to recruit and activate immune cells, TNF-α is decisive for early inflammatory responses that help in clearing infection [38]. Additionally, synergy between the activities of IFN-γ and TNF-α promotes clearing of *Salmonella* from broilers gut [13,38]. Although *B. infantis* BL2416 attached to host cells in lower rate than *L. casei ATTC334* and *B. breve* JCM1192 strains, it promoted IFN-γ and TNF-α production which may contribute to the clearance of infection. Our study demonstrated that the probiotic strain that did not completely clear *S. typhimurium* infection still appeared to significantly lower its counts in the cecal contents. The ability of *B. infantis* BL2416 to increase the immune factors might be mediated via toll-like receptor-regulated signaling pathways that play a role in early events of the innate immunity and modulate pathogen-induced inflammation [39]. In this context, *B. infantis* has been reported to increase the production of Th1-related cytokines, including IFN-γ in mice [40]. However, the exact mechanism needs further studies to be experimentally addressed.

The clinical signs (inappetence, depression, ruffled feathers, and pasty diarrhea) and mortalities observed in birds infected with *S. typhimurium* alone those treated *B. longum* Ncc2785 were consistent with the findings of previous studies [3,4]. All the deaths occurred from day 3 to 7 post challenge with *S. typhimurium*. Birds treated with *B. breve* JCM1192, *L. casei* ATTC334 or *B. infantis* BL2416 then infected with *S. typhimurium* did not exhibit clinical signs or mortality due to the competitive exclusion by *B. breve* JCM1192 and *L. casei* ATTC334 [41] or the increased secretion of IFN-γ and TNFα by *B. infantis* BL2416 [38]. Accordingly, previous studies have shown that treatment with probiotics in case of *Salmonella* infection has improved broiler chickens’ body weight gain and FCR [14,42]. Our results also indicated that the treatment of *S. typhimurium*-infected birds with *L. casei* ATTC334, *B. breve* JCM1192 or *B. infantis* BL2416, respectively, improved body weight gain and FCR when compared with *B. longum* Ncc2785 or the non-treated infected birds.

## 5. Conclusions

Our results identified the set of probiotic bacteria that are likely to contribute to the poultry health and participate in modifying the key immune response in birds. Pre-treatment of the chicks with *B. breve* JCM1192, *L. casei ATTC334* and *B. infantis* BL2416 prevented the deleterious effects of acquired *Salmonella* infection. These three probiotics showed an ability to bind to intestinal cells in vitro, and reduced *S. typhimurium* recovery from the cecal tonsils in vivo. In addition, probiotics improved body weight gain and FCR, and prevented all clinical signs and mortality in *S. typhimurium*-infected chicks. The results suggest that competitive exclusion is the mechanism underlying the protective effect of *B. breve* and *L. casei* and the reduced recovery of *S. typhimurium* from cecal tonsils, whereas *B. infantis* BL2416 exerted its protective effects via promoting IFN-γ and TNF-α production.

## Figures and Tables

**Table 1:** Ingredients, composition and nutrient content of starter, grower and finisher diets.

Ingredients (%)	Starter	Grower	Finisher
Corn grains	56.9	61.6	66.4
Soybean meal 48%	34.31	29.71	24.6
Corn gluten meal 60%	3.5	3	3
Soybean oil	1.5	2	2.71
Dicalcium phosphate	1.6	1.37	1.27
Limestone	1.05	1.11	1
Salt	0.22	0.24	0.25
Sodium bicarbonate	0.32	0.37	0.24
Lysine hydrochloride	0.2	0.2	0.15
D.L Methionine	0.1	0.1	0.08
Premix	0.3	0.3	0.3
Nutrients content			
Metabolizable energy (K Cal/kg)	3050	3120	3150
Crude protein %	23.12	21.02	19.01
Crude fat %	4.01	4.8	5.5
Ash	6.1	5.5	5.0
Acid detergent fiber %	4.51	4.34	4.3
Calcium %	0.97	0.92	0.86
Available phosphorus %	0.45	0.4	0.38

**Table 2:** The group of chicks treated *S. typhimurium* and different probiotic strains.

Groups	Treatment
Control	Inoculated orally with saline.
*S. typhimurium*	Inoculated orally with *S. typhimurium*.
*B. breve* JCM1192 + *S. typhimurium*	Inoculated orally with *B. breve* JCM1192 and *S. typhimurium*.
*L. casei* ATTC334 + *S. typhimurium*	Inoculated orally with *L. casei* ATTC334 and *S. typhimurium*.
*B. longum* Ncc2785 + *S. typhimurium*	Inoculated orally with *B. longum* Ncc2785 and *S. typhimurium*.
*B infantis* BL2416 + *S. typhimurium*	Inoculated orally with *B infantis* BL2416 and *S. typhimurium*.

**Table 3:** *S. typhimurium* recovered from the cecal tonsils and cecal contents of chicks treated with different probiotic strains.

Group	*S. typhimurium*-Positive Cecal Tonsils/Total Cecal Tonsils (%)	*S. typhimurium*-Cecal Recovery (×10^4^)
*S. typhimurium*	10/10 (100%)	230.0 ± 4.14
*B. breve* JCM1192 + *S. typhimurium*	2/10 (20%) *	26.4 ± 8.067 ***
*L. casei* ATTC334 + *S. typhimurium*	1/10 (10%) *	17.18 ± 3.45 ***
*B. longum* Ncc2785 + *S. typhimurium*	9/10 (90%)	179.03 ± 7.81
*B infantis* BL2416 + *S. typhimurium*	3/10 (30%)	22.61 ± 6.65 ***

Data are mean ± SD. * *p* < 0.05 and *** *p* < 0.001 versus *S. typhimurium*.
